# Impact of Early Nutrition Interventions on the Growth and Development of Preterm Infants: A Narrative Review

**DOI:** 10.7759/cureus.54888

**Published:** 2024-02-25

**Authors:** Mamdouh Alanazi, Mohammed A Altawili, Ahmed I Khayyal, Abdulrahman S Alahmari, Asmaa Abdullah Alhakami, Abdulrahman Mohammed A Alshehri

**Affiliations:** 1 Pediatric Cardiology, Maternity and Children Hospital, Tabuk, SAU; 2 General Practice, Alaziziyah Primary Health Care Center, Tabuk, SAU; 3 Pediatrics, King Abdulaziz University Medical Services Center, Jeddah, SAU; 4 General Practice, Bisha Maternity and Children Hospital, Bisha, SAU; 5 Pediatrics, Faculty of Medicine, University of Tabuk, Tabuk, SAU; 6 General Practice, Khamis Mushayt General Hospital, Abha, SAU

**Keywords:** rop, retinopathy of prematurity, prematurity, enteral feeding, development, neurodevelopment, growth, early nutrition interventions, premature babies, preterm birth

## Abstract

Preterm birth remains a significant global health concern as it can lead to various health complications and long-term developmental challenges. Early nutrition intervention plays a crucial role in optimizing the growth, development, and overall health outcomes of premature infants. This review aims to summarize and analyze the existing literature regarding the effect of early nutrition interventions on premature babies. A comprehensive search was conducted through various electronic databases, including PubMed, Scopus, and Google Scholar, focusing on nutrition interventions specifically targeting premature infants.

The review highlights the benefits of early nutrition interventions, including enteral and parenteral feeding, human milk, and the provision of specific nutrients. These interventions have been shown to enhance growth rates, promote neurodevelopmental outcomes, reduce the incidence and severity of retinopathy of prematurity (ROP), reduce the risk of infection, and improve overall morbidity and mortality rates in premature babies. Overall, the findings from this review suggest that early nutrition interventions have a positive impact on the health and developmental outcomes of premature babies. However, further research is required to determine the optimal approaches, optimal timing, and long-term effects of various interventions. Collaboration between healthcare providers, researchers, and families is crucial in implementing evidence-based nutrition practices and supporting the growth and development of premature infants.

## Introduction and background

Low birth weight (LBW) is considered a complex syndrome that includes small-for-gestational-age (SGA), preterm birth (before 37 weeks of pregnancy) babies, or a combination of both. LBW is an important barrier to the survival and development of the child and the maintenance of sustainable development goals [1]. According to a systematic analysis done to estimate the LBW regionally, nationally, and worldwide, 20.5 million (14.6%) infants are born with LBW annually, with 48% in South Asia [2]. LBW and/or preterm birth are also important causes of neonatal mortality. In 2015, there were one million deaths in children under the age of five years globally attributed to prematurity [3]. It is found that preterm labor causes breathing, hearing, and feeding problems in babies [4]. It is also associated with vision problems such as retinopathy of prematurity (ROP) [5].

Nutrition is one of the most important factors that affect the health and growth of newborns. Nutritional management of premature infants is a major clinical challenge because optimal feeding and nutrition systems have not been determined despite extensive studies [6]. The critical role of nutrition begins during pregnancy since it alters the developmental status and metabolic pathways of the fetus, neonate, and child, therefore having long-term effects on adult health. So, poor maternal nutritional conditions early in fetal life have both immediate and long-term effects, such as increased risk of noncommunicable diseases (NCDs) and other chronic diseases like obesity, which is a major risk factor for NCDs [7-9]. In this review, we will summarize and analyze the existing literature regarding the effect of early nutrition intervention on the development and growth of preterm infants.

## Review

Methods

Searches were conducted on the National Institute of Health's PubMed, Scopus, MEDLINE, and WoS databases between 2012 and 2024, using the phrases ("preterm birth" OR "premature babies" OR "Prematurity" OR "Premature Birth" OR "Preterm baby" OR "Premature baby") AND ("early intervention" OR "early nutrition interventions" OR "breast feeding" OR "Early Aggressive Nutrition") AND ("Retinopathy Of Prematurity" OR "ROP" OR "Immunity" OR "Gut Microbiome" OR "Respiratory System" OR "Preterm Brain" OR "birth outcomes" OR "linear growth"). Articles were screened based on their relevance to explore the impact of early nutrition interventions on the growth and development of preterm infants at the discretion of the principal researcher. No limitations were placed on the publication years and study designs.

Impact of early nutrition intervention on birth outcomes and 24 months of age linear growth

Stunting (length-for-age z score ≤2 standard deviations) and LBW remain major parameters that are used as indicators of children's survival and development as well as the accomplishment of sustainable development goals [1]. While stunting is a serious problem worldwide, its prevalence in India surpasses the average for the Asia region. The rate among children under five years is 37.9%, which is higher than the rate for developing countries (8.9%) [10]. The majority of stunting happens in the first two years of life, but its effect lasts a lifetime. So, early intervention starting from the preconception period up to the first two years of life is very important for the lives of children and adults [7,11,12].

A randomized clinical trial was conducted to assess if the birth outcomes and linear growth at 24 months of age would be affected by the integrated and simultaneous delivery of health, nutrition, water, sanitation, and hygiene (WaSH), and psychosocial care interventions [13]. The trial found that preconception, pregnancy, and early childhood intervention programs dramatically reduce stunting and LBW at two years of age. When two years old, the group receiving pregnancy and early childhood interventions alone experienced a significant decrease in the proportion of stunted babies, but the proportion was smaller than the group that received interventions at prenatal, pregnancy, and early childhood periods (incidence rate ratio 0.51, 98.3% CI 0.38 to 0.70 vs. 0.96, 98.3% CI 0.71 to 1.29). This means that both interventions during the prenatal period and the pregnancy and early childhood periods are very important due to their significant effect on the birth and 24-month outcomes. However, the intervention during the preconception period only has a significant impact on birth outcomes, but 24-month results are unaffected. Another randomized clinical trial published in 2017 [14] found that high-dose amino acid starting at birth is not recommended because it is not associated with improving growth or neurodevelopmental outcomes.

Impact of early aggressive nutrition on retinal development in premature infants

**Figure 1:**
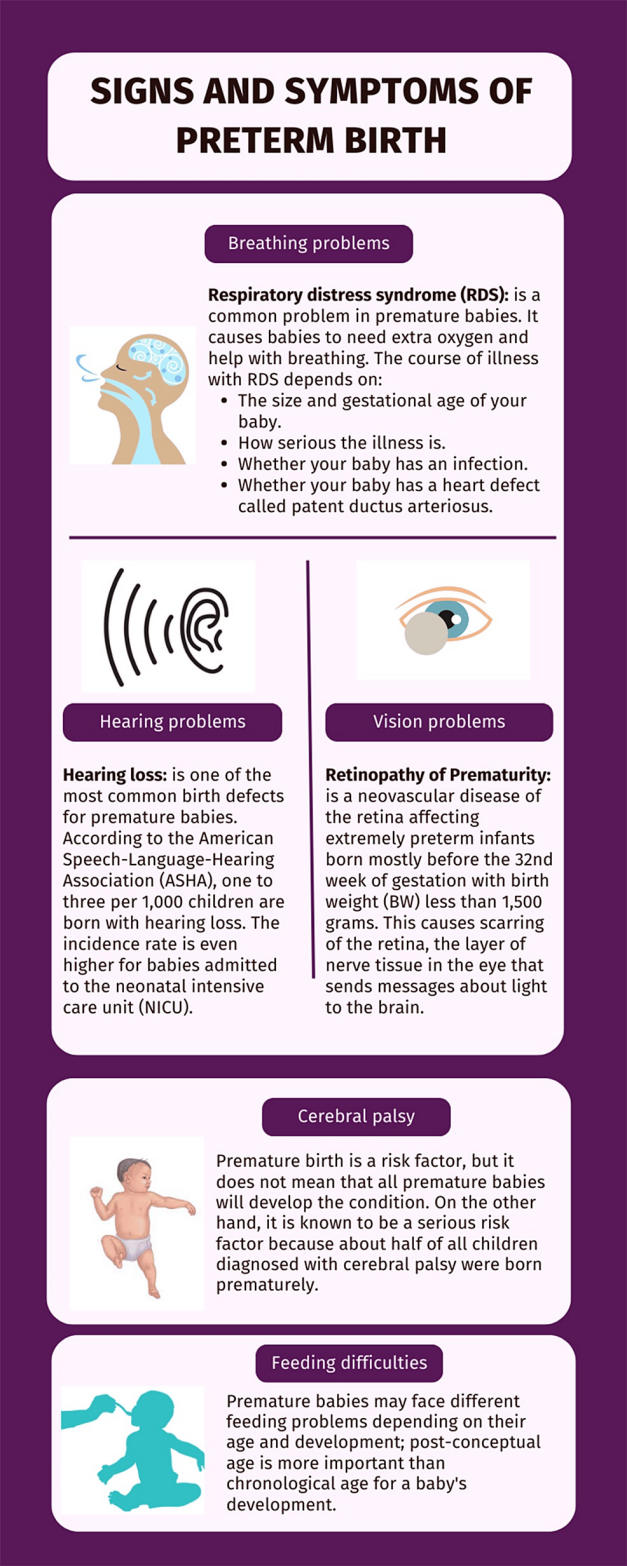
Signs and symptoms of a preterm baby Image credits: All the authors of this study.

As we mentioned before, preterm infants are at an increased risk of developing several complications such as breathing, hearing, and feeding problems [4], it is also associated with vision problems such as ROP (Figure 1) [5].

Retinopathy of prematurity (ROP) is a neovascular retinal illness that primarily affects extremely preterm newborns with birth weights (BW) of less than 1,500 grams who are born before the 32nd week of gestation [5]. Premature birth causes an interruption in normal retinal development, which leads to the start of ROP. Blindness may result from retinal detachment, scarring of the retina, and abnormal retinal vascularization. ROP is still the most common cause of juvenile blindness and visual impairment, despite the slow improvement in neonatal care and the rising number of severely preterm newborns that survive. It can result in up to 101.6 thousand cases of visual impairment, including 27.5 thousand severe, 49.1 thousand moderate, and 25.0 thousand blindness cases [15]. Most cases of ROP occur in middle-income countries, where approximately one-third of very LBW infants develop some form of ROP [16], and its incidence rises when BW and gestational age (GA) decrease [17,18].

Retinal development begins in the fourth month of pregnancy and lasts until the 40th week of gestation. Thus, the retinal vasculature of an extremely premature baby is inadequate at birth. The optic disk's center is where retinal blood vessels expand outward to reach the ora serrata, with the periphery remaining avascular until around the 32nd week of pregnancy. The relative hypoxia that results from this condition in the retinal tissue serves as a stimulus for hypoxia-inducible factors, which in turn cause the transcription of angiogenic genes to create growth factors including vascular endothelial growth factor (VEGF), its analog placental growth factor (PIGF), and the proangiogenic EPO. VEGF triggers the formation of new endothelial cells (vasculogenesis) and the subsequent budding of new blood vessels from these cells (angiogenesis) in response to this wave of physiological tissue hypoxia [19]. IGF-1 regulates VEGF-mediated vascularization and is necessary for the survival and growth of the newly formed endothelial cells. The availability of amino acids and total energy intake control the production of IGF-I. Thus, it would be reasonable to conclude that IGF-1 levels are a good indicator of nutritional status. When there is enough nutrition available during the third trimester, the levels often increase two or three times, which encourages protein accretion and rapid fetal growth [20,21].

Several studies have investigated the relationship between early nutrition and the reduction of the risk of ROP. A study published in Clinical Nutrition in 2019 [22] found that every 10 kcal/kg/d increase in energy intake between days 7 and 27 was linked with a 6% lower incidence of any grade of ROP in multivariable models. Additionally, a meta-analysis published in 2023 evaluated the effects of early enteral and parenteral nutrition on ROP to summarize the recent findings from various sources to give an insight into the relationship between dietary practices and ROP risks and concluded that early oral and intravenous nutrition has a major effect on a child's ROP severity and progression [23].

While these findings suggest that early nutrition intervention may have a protective effect against ROP, it is important to note that further research is still needed to fully understand the optimal nutritional strategies to prevent ROP in premature infants. Consulting with healthcare professionals is crucial to ensure individualized care for each premature infant regarding nutrition and ROP prevention.

Impact of early nutritional intervention on preterm brain

When a newborn infant undergoes a preterm birth, he/she can develop some effects on the brain such as intraventricular hemorrhage (IVH), a condition characterized by bleeding in and around the ventricles of the brain. This can cause damage to the developing brain and lead to long-term complications [24]. They also may have periventricular leukomalacia (PVL), which is characterized by softening or death of white matter near the ventricles. It can disrupt the flow of information in the brain, resulting in motor and cognitive impairments [25]. Intellectual disability is another problem that occurs with some preterm infants later in life, which can affect their cognitive abilities, academic performance, and overall functioning [26]. Early intervention, specialized care, and supportive environments can help minimize some of these challenges and improve long-term outcomes for preterm babies [27].

Early nutritional intervention has been found to have a significant impact on the brain development of preterm infants as neurodevelopment is greatly aided by certain nutrients, particularly macronutrients and micronutrients. Early and aggressive feeding has shown promising results, as has the recognition of glucose as an essential source of energy for the developing brain. In addition, it was found that adequate nutrition plays a vital role in promoting brain growth in preterm infants because it provides the necessary nutrients and energy for brain cells to develop, myelinate, and form connections. So, a well-balanced diet, including macronutrients (protein, carbohydrates, and fats) and micronutrients (vitamins, and minerals), is crucial for optimal brain development in premature babies [28]. Cognitive development is also affected by nutritional intervention in the early stages of life. Studies have shown that providing a higher caloric intake and specific nutrients like docosahexaenoic acid (DHA) and arachidonic acid (ARA) can enhance cognitive function, attention, and memory in these infants [29]. Furthermore, another study looked at the connection between nutrition and neurodevelopment in premature babies and emphasized the value of customized dietary plans and all-encompassing nutrient strategies to support neurodevelopment in premature babies [30]. It concluded that the role of human milk in early brain maturation underscores its potential as the gold standard for the nutrition of premature infants. The authors also covered how certain nutrients, such as macronutrients (glucose, amino acids, and lipids) and micronutrients, might support neurodevelopment. DHA and other long-chain polyunsaturated fatty acids aid in the maturation of the brain. Probiotics and human milk oligosaccharides are also beneficial for neurodevelopment through the gut-brain axis [30].

In summary, early nutritional intervention in preterm infants has a profound impact on brain development and cognitive function. Providing a well-balanced diet rich in essential nutrients is crucial for maximizing developmental outcomes and reducing the risk of neurodevelopmental disorders.

Impact of early nutritional intake on preterm respiratory system

Early nutritional intake plays a crucial role in the development and functionality of the respiratory system in preterm infants due to its essential role in optimal lung growth and maturation [31]. Appropriate nutrition, including proper protein and calorie intake, is important for enhancing the lung development of preterm infants, resulting in improved respiratory function [32]. It also protects against certain respiratory disorders [33]. For example, providing appropriate amounts of specific nutrients, such as antioxidants, may reduce the risk of bronchopulmonary dysplasia (BPD), a common respiratory complication in preterm infants [34]. Furthermore, a meta-analysis on breastfeeding and BPD discovered that when premature neonates were exclusively breastfed as compared to formula-fed, the incidence of BPD decreased [35].

Because of the important role of nutrition intervention, it is important for healthcare providers to closely monitor the nutritional needs of preterm infants and customize feeding strategies accordingly. Individualized nutrition plans that include appropriate energy, protein, vitamins, and minerals can optimize the respiratory system's development and function, reducing the risk of respiratory complications in these vulnerable infants [31].

Impact of early intervention on the immunity and gut microbiome in preterm infants

The pathophysiology of many problems of preterm infants, including infections, is greatly influenced by the combination of immature organs and the high requirement for invasive treatment and life support measures [36]. Specifically, the immature immune system is significant because several innate and acquired immune system components are shown to be inhibited and limited in their development and function, which accounts for preterm newborns' vulnerability to infectious illnesses [37,38]. Many studies show that premature babies are more susceptible to infections due to their immature immune systems, and the incidence of the infection decreases when the GA increases [39,40].

Different interventions such as optimal nutrition, early skin-to-skin contact, probiotic supplementation, and the avoidance of unnecessary antibiotic use can have a significant impact on the immunity of preterm infants [38,41-43]. Optimal nutrition is critical for the management of preterm infants. It improves survival while decreasing the potential for both short- and long-term morbidities [44]. A study suggested that maternal diet may enhance the immune systems of premature infants [45]. The study found that maternal diet has a profound impact on the immune system of developing fetuses and that breastfed babies have a more robust ability to fight disease, suggesting that even after childbirth, a mother’s diet matters. For instance, studies have examined the benefits of breastfeeding and the introduction of human milk fortifiers, which can improve the immune system by providing essential nutrients and antibodies [46,47].

Furthermore, the administration of probiotics has gained attention in recent years. Probiotics, specifically certain strains of beneficial bacteria, have been found to enhance the immune function of preterm infants, reducing the risk of infections [42]. Probiotic supplementation has been shown to reduce the incidence of necrotizing enterocolitis (NEC) in preterm infants, which is a serious intestinal disease that can compromise their immune system [48,49]. The American Academy of Pediatrics recommends the use of probiotics in preterm infants who are at risk for NEC [48]. However, it is important to note that the FDA has not approved any probiotic product for use as a drug or biological product in infants [50]. Early intervention of probiotics has been shown to alter the gut microbiota of preterm infants [51]. Probiotic supplementation has been associated with a reduction in opportunistic pathogens such as *Weissella*, *Veillonella* spp., and *Klebsiella* as well as potential nosocomial pathogens like *Citrobacter *and *Chryseobacterium* species [52,53].

Overall, studies highlight the importance of strategies such as optimal nutrition, probiotic supplementation, and judicious use of antibiotics. These interventions aim to enhance immune development and promote a healthy gut microbiome, ultimately improving the long-term health outcomes of preterm infants.

Initial nutritional management of the preterm infant

In the initial nutritional management of preterm infants, several key principles are followed to meet their unique nutritional needs. Whenever possible, the first choice for feeding preterm infants is human breast milk. It provides optimal nutrition and immune protection and supports the development of the infant's gut. Additionally, it improves neurodevelopmental outcomes and lowers the risk of infection and NEC, while it may also slow down linear and ponderal growth [54]. It is important to start enteral feeding (feeding through the gastrointestinal tract) as soon as the infant is stable and able to tolerate it. This helps support gut maturation and reduces reliance on parenteral nutrition (intravenous feeding) [55-57]. If the mother's breast milk has not yet "come in," it is advised to be fed pasteurized human donor milk. When a baby has anemia, iron supplements can be given directly, or the mother's diet can be strengthened with foods high in iron [58]. Adequate protein intake is crucial for growth in preterm infants. Breast milk or formula should provide a sufficient amount of protein to support optimal growth. The protein content may be adjusted based on the infant's individual needs and growth [59].

In preterm infants, the gastrointestinal system is indeed immature and needs time to develop fully. Feeding advancement is typically done gradually to ensure that the infant's digestive system can handle increasing volumes of milk over time [56,57]. Initially, small amounts of milk are given to preterm infants often in the form of breast milk or fortified preterm formula. The volume of milk is then increased slowly and cautiously, taking into account the infant's tolerance, growth, and any specific medical conditions [60]. Advancing feeds in preterm infants are individualized and closely monitored by healthcare professionals, such as neonatologists, pediatricians, or neonatal nurses. They assess the infant's tolerance to feeding by observing signs such as adequate weight gain, proper functioning of the gastrointestinal system, the absence of feeding-related complications, and the ability to handle increased volumes without issues like reflux or aspiration [61]. Preterm infants may have increased requirements for certain vitamins and minerals. These may be provided through fortified breast milk, preterm formula, or separate micronutrient supplements, as required (Table 1) [62].

**Table 1:** Important nutrient interventions for premature babies and their importance VLBW: Very low birth weight; ROP: Retinopathy of prematurity.

Nutrient intervention	Importance
Iron supplementation [63]	Iron supplementation is crucial for premature babies as they are often born with low iron stores. Iron is essential for the production of red blood cells and plays a key role in brain development. Supplementing iron helps prevent iron deficiency anemia and supports overall growth and development.
Vitamin E supplementation [64]	Hemoglobin concentration was considerably raised by routine vitamin E intervention, but only slightly. Vitamin E raised the risk of sepsis and dramatically decreased the chance of germinal matrix/intraventricular hemorrhage. Other morbidity or death was not significantly impacted by vitamin E. Vitamin E intervention dramatically decreased the incidence of serious retinopathy and blindness in VLBW newborns while increasing the risk of sepsis.
Vitamin D supplementation [65]	All newborns, preterm and full term, should have adequate dietary vitamin D consumption, with a focus on those who are getting human milk. For healthy newborns, the recommended daily total food intake is 400 IU. There are several ways to give babies vitamin D, and parents can choose whatever way they like.
Omega-3 fatty acids [66,67]	Omega-3 fatty acids, particularly DHA (docosahexaenoic acid), are critical for the development of the premature baby's brain and eyes. Supplementing with breast milk or specialized formula containing omega-3 fatty acids supports healthy brain and visual development. The frequency of ROP was reduced with the use of omega-3. The severity of ROP is also significantly reduced with the use of omega-3.

It is important to note that specific feeding strategies may vary based on the individual needs and conditions of each preterm infant. Hence, it is always recommended to consult with a healthcare professional who can provide personalized advice and guidance tailored to the preterm infant's specific case [61].

Recommendation for future research

The following suggestions are aimed at improving the impact of early nutrition interventions on the growth and development of preterm infants:

Implement long-term studies that track preterm infants' growth and development from infancy to early childhood. This would offer valuable insights into the long-term benefits associated with early nutrition interventions.

Develop personalized nutrition plans for preterm infants based on their specific needs and health conditions. This could involve individualized assessments, including genetic profiling and metabolic evaluations, to optimize nutrition interventions for each infant.

Enhance the fortification of breast milk for preterm infants. Research and develop methods to increase the nutrient content, especially for specific nutrients crucial for preterm infants' growth and brain development, such as omega-3 fatty acids, iron, and vitamin D.

Establish and expand breast milk banking programs to ensure a steady supply of donor milk for preterm infants. This can help bridge the nutritional gap for infants whose mothers are unable to provide sufficient breast milk.

Explore and develop innovative feeding techniques to enhance the delivery of nutrition for preterm infants. This could include techniques such as orogastric or nasogastric tube feeding as well as novel approaches like transpyloric tube placement or direct oral feeding.

Continuously evaluate and refine the nutrient composition of specialized formulas and fortifiers used for preterm infants. Regularly assess the impact of these compositions on growth, neurodevelopment, and long-term health outcomes.

Encourage family involvement and education in the care and feeding of preterm infants. Providing support, resources, and education to parents can empower them to actively participate in their child's nutrition and development.

Foster collaboration between healthcare professionals, nutritionists, psychologists, and developmental specialists to comprehensively address the needs of preterm infants. This interdisciplinary approach ensures a holistic and integrated approach to early nutrition interventions.

Raise awareness among healthcare providers, parents, and the general public about the critical role of early nutrition in preterm infants' growth and development. Conduct educational campaigns and provide accessible resources to disseminate evidence-based information.

Allocate resources to facilitate further research in this field, including clinical trials, observational studies, and population-based studies. Continued research and evidence generation are essential to refine and optimize early nutrition interventions for preterm infants.

These recommendations can serve as a starting point for future improvements in the field of early nutrition interventions for preterm infants.

## Conclusions

In conclusion, the findings of the review suggest that early nutrition interventions play an essential role in the growth and development of preterm infants. Proper nutrition during the early stages of life is essential for achieving optimal growth, neurodevelopment, and overall health outcomes in these vulnerable infants. Human milk is a preferred feeding option for preterm infants. Human milk provides numerous benefits, including immunological protection, improved gut health, and better cognitive development compared to artificial milk.

Early initiation of enteral feeding, such as breast milk, and the use of specific nutritional supplements were also found to have positive effects on growth and neurodevelopment. While the existing literature provides valuable insights into the effect of early nutrition interventions on preterm infants, further research such as long-term follow-up studies and standardized protocols is required to establish evidence-based guidelines for early nutritional management in this population.
